# Rape in Texas—Castration the Only Remedy

**Published:** 1895-03

**Authors:** 


RAPE IN TEXAS—CASTRATION THE ONLY REMEDY.—By an official report, published recently in the *Statesman*, it is shown that in 1893 and 1894 there were 155 indictments for rape in Texas, and 37 convictions [the number of executions not stated]. It was stated in the report that the number, 155, was an increase of 20 cases over the previous two years! There must have been, then, in 1891-2, 135 cases, an increase of about 15 per cent.

That the crime should so greatly increase in face of the fact that death is the penalty, looks a little remarkable; but when it is seen that only 37 are convicted out of 155 indictments, the wonder ceases. May we not find here an explanation of why, now and then, "Judge Lynch" takes a hand? If the death penalty by hanging does not lessen the crime; if the law is so loosely administered that three-fourths of the offenders escape conviction, is it any wonder that the people now and then take the matter in hand? Yet it seems that even lynching will not stop it. What then are the people of Texas to do?

There is at present before the legislature a bill to make castration a penalty for rape, and we learn that the Senate committee have reported favorably upon it. We learn, also, that a popular Senator has strongly objected to it, on the grounds that it is "a cruel and unusual punishment" and that the Constitution prohibits such punishment. Away with all such maudlin sentiment and infinitesimal hair splitting. If the castration of a buck negro who has raped and murdered a young child, is cruel and unusual, what would the Senator call the fate of that child? The bill ought to pass by all means.

The people make the laws. By observation and experience it is ascertained what evils are to be met and punished, and a law is constructed to meet each offense. But there are crimes, new and unusual, and surpassing in horror any known heretofore to civilized man, and for such crimes no adequate punishment has been provided. The crime of the monster Smith, at Paris, and of Nichols, in this county, were more than rape. The former was too horrible even to be classified; it was rape, plus murder, plus everything diabolic that can be conceived. There is on our statutes no law to punish such crime; it was not dreamed of when the penal code was made. Castration, even, would not meet the requirements of such a case. Are the people to be blamed then, if, in their frenzy and despair they forgot, awhile, their humanity, and inflicted the only punishment at all adequate to the case?

But the law should aim at something more than punishment; its chief object should be the prevention of crime, and it is patent to the most casual observation that hanging has utterly failed to diminish crime. We want something that will, and, at the same time, fulfill the other ends of jurisprudence,—protection of society—prevention of repetition, and—of greater importance, perhaps,—that will stop the breeding of criminals.

Castration, or castration plus penitentiary, will, in our judgment, fill all the requirements; it will lessen the crime of rape. Let every doctor who reads this, write to his representative, requesting him to vote for the bill.

